# Effects of Exogenous Auxin on Mesocotyl Elongation of Sorghum

**DOI:** 10.3390/plants12040944

**Published:** 2023-02-19

**Authors:** Chang Liu, Ziqing Yao, Bing Jiang, Wenbo Yu, Yu Wang, Wenhui Dong, Yutong Li, Xiaolong Shi, Chunjuan Liu, Yufei Zhou

**Affiliations:** 1 College of Agronomy, Shenyang Agriculture University, Shenyang 110866, China; 2 Jinzhou Academy of Agricultural Sciences, Jinzhou 121006, China

**Keywords:** *Sorghum bicolor* L. Moench, mesocotyl length, energy metabolism, plasma membrane acidification, expansin

## Abstract

The length of sorghum mesocotyl plays a vital role in seed emergence from the soil, which is the foundation of healthy growth. In this study, we aimed to understand how exogenous auxin (IAA) promoted mesocotyl elongation of sorghum and its physiology mechanism. The results presented that exogenous IAA significantly promoted mesocotyl elongation in MS24B (short mesocotyl inbred line) by increasing the cell length, while with extra exogenous NPA (IAA inhibitor) application, the mesocotyl length presented a significant short phenotype. In Z210 (long mesocotyl inbred line), exogenous IAA had a slight effect on mesocotyl length elongation, while the NPA treatment decreased the mesocotyl length considerably. In MS24B, IAA treatment increased the activity of amylase to degrade starch to soluble sugar, and the activity of hexokinase was improved to consume the increased soluble sugar to offer more energy. The energy will help to increase the activity of PM H^+^-ATPase and the expression of expansin-related genes, which ultimately will promote the acidification of the plasma membrane in MS24B for cell elongation. Overall, the exogenous IAA functioned on the activation of energy metabolism, which in turn, inducted the acidification of the plasma membrane for mesocotyl elongation.

## 1. Introduction

Sorghum (*Sorghum bicolor* L. Moench) is an important food, feed, fiber, and biofuel crop worldwide. It provides nutrients and calories for much of the world’s population. “Early development” is a term used to describe plants that can grow fast at an early stage. The early development varieties normally have good competitiveness with weeds and could effectively improve nutrient use efficiency. However, there was always a low emergence rate of sorghum because of improper cultivation methods or the unstable sowing depth. Deep sowing is the priority barrier for seed emergence. It is a considerable problem that affects sorghum’s early development and growth. Researchers claimed that mesocotyl length is one of the most important factors in sorghum seed emergence. Mesocotyl is the organ located between the coleoptilar node and the basal part of the seminal root in young seedlings [1,2]. It is an important channel connecting the seed and the up ground. During the germination stage, the breakthrough of the shoot tip from the soil mainly depends on the elongation of mesocotyl. Longer mesocotyl has good emergence performance, which in turn, helps to start photosynthesis early, then finally has positive effects on the whole growth process. According to the research, the length of rice mesocotyl is positively related to the growth situation in the germination stage. The seedling rate of long mesocotyl lines was significantly higher than that of the medium and short lines [3,4]. In maize, mesocotyl elongation is beneficial for emergence, and it is the reason for the good performance of deep sowing [5]. 

The essence of mesocotyl elongation is the extension and division of mesocotyl cells, in which cell extension growth plays a decisive role [6,7]. Li et al. [8] identified that the increase in cell length contributed more to the elongation of the mesocotyl, and the promoting effect of the number of cells on the elongation of the mesocotyl only occurred in the early stage of elongation, while the effect of cell extension on the mesocotyl elongation is throughout the entire period of mesocotyl development. However, the extension is restricted by the cell wall. The relaxation and extension of the cell wall is the key to mesocotyl elongation [9]. PM H^+^-ATPase is a type of proton pump, which can be induced by IAA to pump H^+^ out of the cell and activate cell wall relaxase to promote cell wall relaxation, which in turn, offers the extension space for mesocotyl cell growth [10,11]. In other words, the apoplast pH regulates cell wall composition and elongation. The acidification of the apoplast leads to cell wall relaxation, which is promoted by expansins to bind to the hydrophilic region and release the covalent bond. Expansins are a family of proteins that catalyze long-term extension of isolated cell walls. It is reported to play a vital role as a modulator of cell wall extensibility, implicating cell elongation and plant growth processes [12,13]. For example, Lee et al. [14] claimed that the expansin gene *GmEXP1* is correlated with root cell elongation in soybean. Another expansin gene *OsEXPA10* is reported to be associated with root cell elongation in rice [15]. Furthermore, the reaction of PM H^+^-ATPase is based on the energy supplement from the seeds. The degradation of starch in the endosperm is the major energy source for mesocotyl elongation. In sorghum, the process of starch degradation needs amylase enzymes such as α-amylase, β-amylase, limit dextrinase (LDA), and maltase [16]. The α-amylase plays a remarkable role in catalyzing starch granules degradation, and its activity is positively associated with germination rate and following growth [17,18,19]. 

Auxin plays a crucial role in many aspects of plant growth and development [20]. Previous studies have shown that exogenous auxin can stimulate organ elongation. In Arabidopsis, IAA application could elongate hypocotyl [21]. According to the research of Cao et al. [22], different concentrations of IAA application will elongate various lengths of mesocotyl. With the IAA concentration of 2 μL/L, the rice mesocotyl promotes the elongation by 0.085 cm. In addition, similar results proved that the exogenous IAA could increase the length of etiolated rice seedlings for 2 days after germination in darkness [23]. 

So far, few studies have been reported on the effect of exogenous IAA regulating the mesocotyl of sorghum. It is meaningful to understand how the effect of exogenous IAA is on the elongation of mesocotyl length, whether there is a variation in response to exogenous IAA in lines with different mesocotyl lengths, and which physiology pathways take part in the mesocotyl elongation process. In this research, we identified the function of exogenous IAA application on sorghum mesocotyl length and explained how IAA regulates the length of sorghum mesocotyl, including the analysis of the variation encompassing in morphology, membrane acidification pathway, and energy metabolism pathway under IAA treatment. 

## 2. Results 

### 2.1. The Effect of IAA Treatment to Mesocotyl Length of Different Sorghum Lines

**Figure 1:**
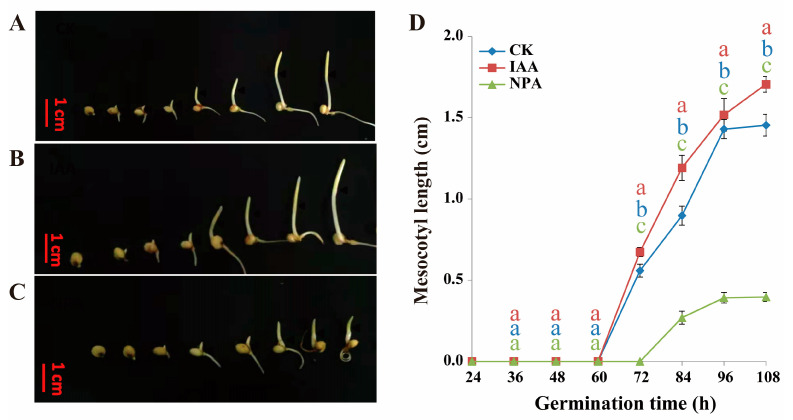
Mesocotyl length at different germination time in line MS24B. (**A**) Mesocotyl morphology phenotype at 24 h, 36 h, 48 h, 60 h, 72 h, 84 h, 96 h, and 108 h of line MS24B; (**B**) Mesocotyl morphology phenotype at each timepoint of line MS24B with exogenous IAA application; (**C**) Mesocotyl morphology phenotype at each timepoint of line MS24B with exogenous IAA and NPA application; (**D**) Line graph of mesocotyl length of three treatments at each timepoint of MS24B. All data present the mean ± SD of three independent experiments. Lowercase letters indicate statistical differences (*p* ≤ 0.05) among treatments.

**Figure 2:**
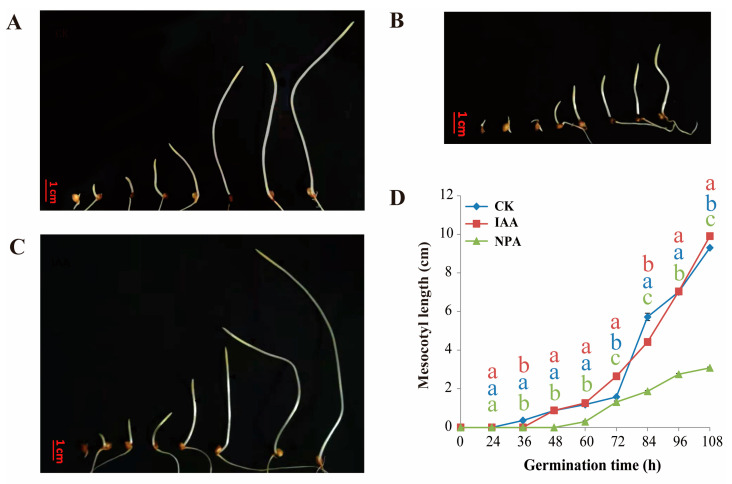
Mesocotyl length at different germination time in line Z210. (**A**) Mesocotyl morphology phenotype at 24 h, 36 h, 48 h, 60 h, 72 h, 84 h, 96 h, and 108 h of line Z210; (**B**) Mesocotyl morphology phenotype at each timepoint of line Z210 with exogenous IAA and NPA application; (**C**) Mesocotyl morphology phenotype at each timepoint of line Z210 with exogenous IAA application; (**D**) Line graph of mesocotyl length of three treatments at each timepoint of Z210. Lowercase letters indicate statistical differences (*p* ≤ 0.05) among treatments.

Previous experiments proved that sorghum variety Z210 had relatively longer mesocotyl compared to that of sorghum variety MS24B. Specifically, short mesocotyl line MS24B was more sensitive to IAA, which presented longer mesocotyl after application of IAA compared with CK treatment, while the exogenous application of NPA (IAA inhibitor) led to a shorter mesocotyl. In Figure 1, the mesocotyl started to grow at 60 h after germination in IAA and CK treatment in line MS24B. In contrast, it delayed 12 h to begin the growth in the treatment of NPA. In line Z210, IAA and CK treatment had nearly the same trend in the mesocotyl growth, which started at the same time point of 24 h after germination. It still presented a delayed start of growth time at 48 h after germination in the treatment of NPA for the line of Z210 (Figure 2). Overall, the exogenous application of IAA can increase the length and earlier excitation of the growth of sorghum mesocotyl.

### 2.2. The Elongation of Mesocotyl by IAA Treatment Depending on the Increase of Mesocotyl Cell Length

**Figure 3:**
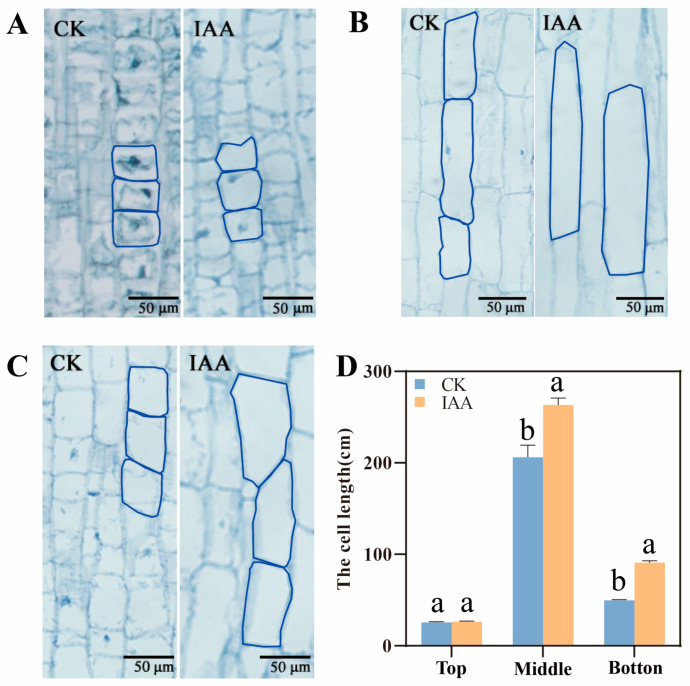
Cell length of different parts of mesocotyl in line MS24B. (**A**) The up part of mesocotyl cell in CK and IAA treatment under the vision of stereomicroscope in MS24B; (**B**) The middle part of mesocotyl cell in CK and IAA treatment under the vision of stereomicroscope in MS24B; (**C**) The down part of mesocotyl cell in CK and IAA treatment under the vision of stereomicroscope in MS24B; (**D**) The cell length statistics of different parts of mesocotyl in MS24B. Lowercase letters indicate statistical differences (*p* ≤ 0.05) among treatments.

**Figure 4:**
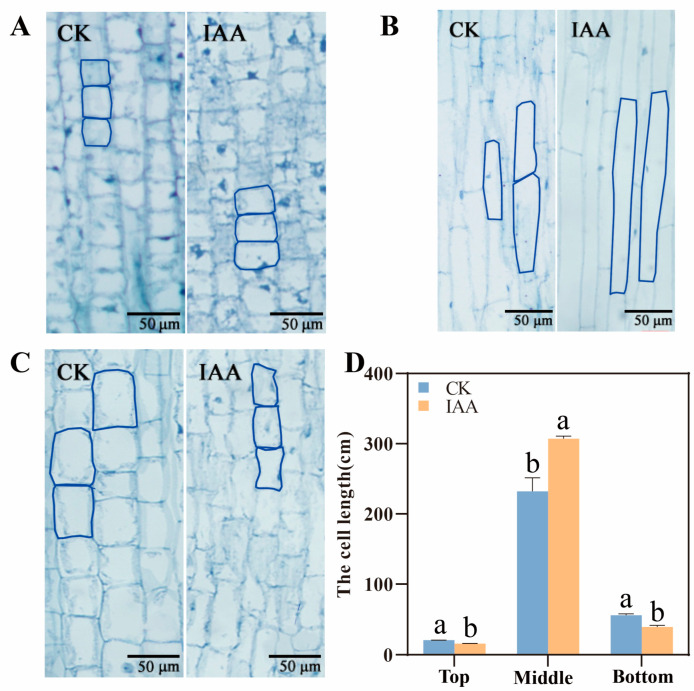
Cell length of different parts of mesocotyl in line Z210. (**A**) The up part of mesocotyl cell in CK and IAA treatment under the vision of stereomicroscope in Z210; (**B**) The middle part of mesocotyl cell in CK and IAA treatment under the vision of stereomicroscope in Z210; (**C**) The down part of mesocotyl cell in CK and IAA treatment under the vision of stereomicroscope in Z210; (**D**) The cell length statistics of different part of mesocotyl in Z210. Lowercase letters indicate statistical differences (*p* ≤ 0.05) among treatments.

In order to understand the effect of mesocotyl cell shape on mesocotyl length, a paraffin section was applied to observe the upper, middle, and down parts of mesocotyl cells. The results in line MS24B showed that the IAA treatment mainly increased the middle and lower part of mesocotyl. There was a 27.7% and 83.2% increase in cell length in the middle and lower part of mesocotyl under IAA treatment, respectively (Figure 3). The results in line Z210 presented that the cell length in the middle part of mesocotyl increased dramatically. It had a 32.4% boost (Figure 4). Overall, the exogenous application of IAA promoted the middle and lower part of mesocotyl length growth in short mesocotyl variety MS24B while it increased only the middle part of mesocotyl length in long mesocotyl variety Z210. 

### 2.3. IAA Application Promote Energy Metabolism Process

**Figure 5:**
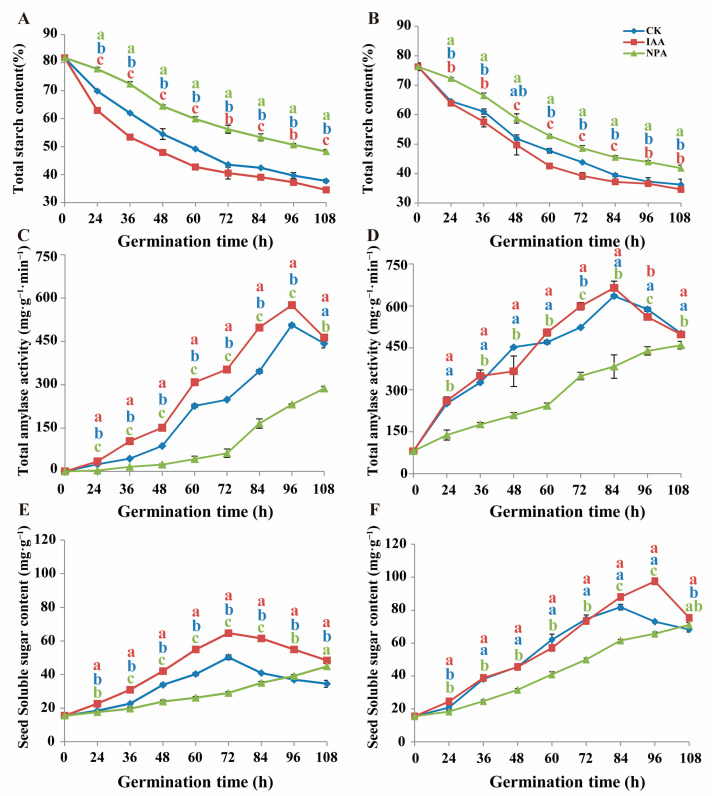
Effects of IAA and NPA treatment on starch content, total amylase activity, and seed soluble sugar of different sorghum inbred lines during germination. (**A**) Total starch content changes from 0 h to 108 h after germination in MS24B; (**B**) Total starch content changes from 0 h to 108 h after germination in Z210; (**C**) Total amylase activity changes from 0 h to 108 h after germination in MS24B; (**D**) Total amylase activity changes from 0 h to 108 h after germination in Z210; (**E**) Soluble sugar content changes from 0 h to 108 h after germination in MS24B; (**F**) Soluble sugar content changes from 0 h to 108 h after germination in Z210. Lowercase letters indicate statistical differences (*p* ≤ 0.05) among treatments.

The starch content and total amylase activity were measured from 0 h to 108 h after germination. In line MS24B, all three treatments showed a downward trend in the total starch content (Figure 5A). The decomposition rate of the IAA treatment was higher than that of CK, while that of the NPA treatment was lower than that of CK treatment. Specifically, the total starch content of the NPA treatment was 50.65%, while the CK and IAA treatments were 39.67% and 37.25%, respectively. The total amylase activity in three treatments was consistent with the result of total starch content. The results presented that the total amylase activity in the IAA treatment was the highest following the CK treatment. The NPA treatment had the lowest total amylase activity (Figure 5C). In line MS24B, the content of soluble sugar presented a rising trend from 0 h to 72 h, followed by a fall trend from 72 h to 108 h for both CK and IAA treatment. The soluble sugar content of the IAA treatment was all the way higher than that of CK. They both reached the highest soluble sugar content in seeds at 72 h after germination, with a content of 50.28 mg/L and 64.68 mg/L in CK and IAA treatment, respectively. The soluble sugar content for the NPA treatment was lower than that of CK from 0 h to 96 h (Figure 5E). 

In line Z210, the starch content at each time point was roughly the same for the IAA treatment and the CK treatment, which were all lower than that of the NPA treatment (Figure 5B). In addition, the total amylase activity result matched the total starch content of each treatment. It increased from 0 h to 96 h after germination for both IAA treatment and CK treatment. The highest amylase activity was 664.51 mg/g·min and 635.34 mg/g·min for the IAA treatment and CK treatment, respectively. The amylase activity for the NPA treatment was significantly lower than that of the IAA treatment and CK treatment at all time points (Figure 5D). In line Z210, the trend and content of soluble sugar were approximately the same in CK and IAA treatment from 0 h to 84 h. The soluble sugar content of CK arrived at the top amount (81.68 mg/L) at 84 h. In addition, the soluble sugar content of the IAA treatment reached the largest amount of 97.58 mg/L at 96 h. The NPA treatment also presented a relatively low soluble content than that of CK from 0 h to 108 h (Figure 5F). 

**Figure 6:**
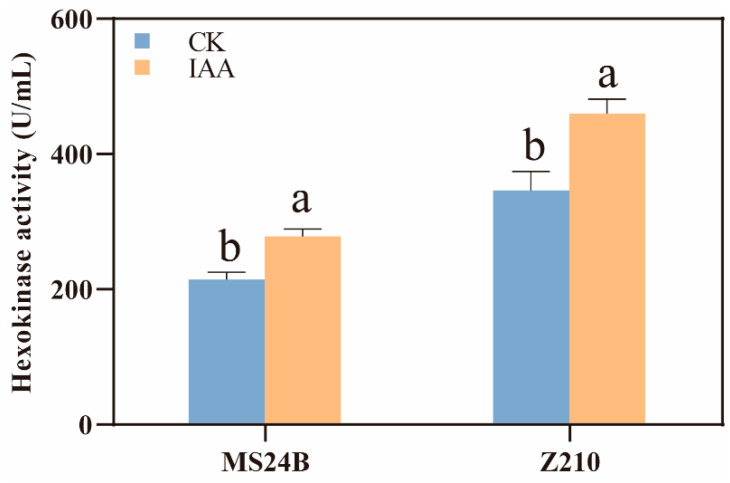
Effects of IAA treatment on hexokinase activity of sorghum mesocotyl. Lowercase letters indicate statistical differences (*p* ≤ 0.05) among treatments.

Hexokinase (HK) is one of the major enzymes in the glycolysis process, and its activity can affect the energy amount for mesocotyl growth. Here we detected the effect of exogenous treatment of IAA and NPA on the activity of HK. In MS24B, the result indicated that the HK activity increased significantly in the IAA treatment compared with that of CK at 96 h after germination (*p* < 0.05). The NPA treatment also increased HK activity considerably (*p* < 0.05). In Z210, the HK activity in IAA treatment was remarkably higher than that in CK. Meanwhile, there was no significant difference between the NPA and CK treatment in HK activity (Figure 6). 

### 2.4. IAA Application Promote Mesocotyl Length by Membrane Acidification

**Figure 7:**
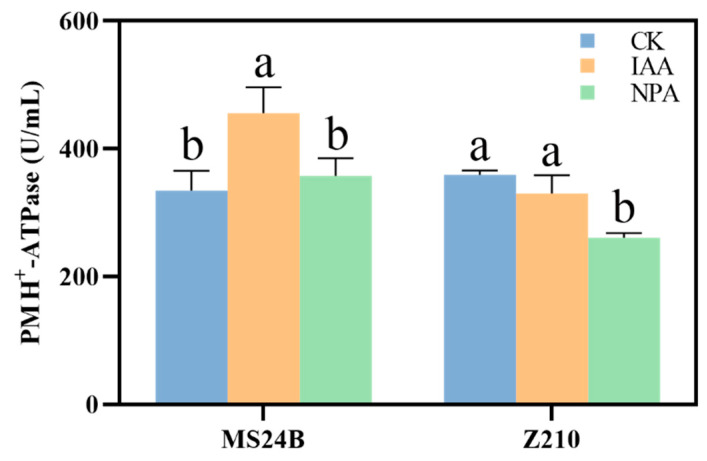
The effect of IAA and NPA on PM H^+^-ATPase activity in line MS24B and Z210. Lowercase letters indicate statistical differences (*p* ≤ 0.05) among treatments.

PM H^+^-ATPase plays a vital role in cell elongation in terms of its function in maintaining cell pH and membrane acidification. According to the result, in the mesocotyl of MS24B, the activity of H^+^-ATPase reached 456.00 U/mL after the IAA application at 96 h after germination, which was 37.7% higher than that of CK (with a value of 331.08 U/mL). The H^+^-ATPase activity of NPA treatment (357.79 U/mL) had no remarkable difference compared with that of CK at 96 h after germination (Figure 7).

The IAA treatment to line Z210 showed a lower but not significant activity of H^+^-ATPase with the value of 330.34 U/mL in comparison with that of CK (359.28 U/mL) at 96 h after germination in mesocotyl. However, a sudden decrease in the activity of H^+^-ATPase was found in the treatment under NPA treatment with a value of 260.78, which was 27.4% less than that of CK (Figure 7).

**Figure 8:**
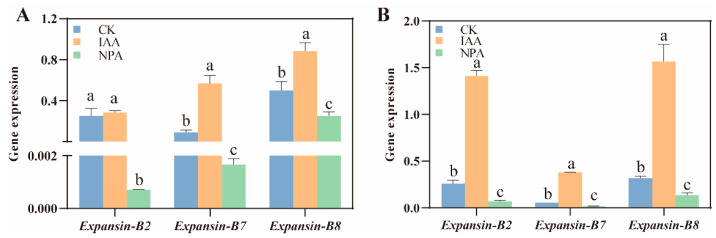
The effect of IAA and NPA treatment to the gene expression of *Expansin-B2*, *Expansin-B7,* and *Expansin-B8.* (**A**) Relative expression of these three genes in line MS24B; (**B**) Relative expression of these three genes in line Z210. Lowercase letters indicate statistical differences (*p* ≤ 0.05) among treatments.

Expansin is one of the proteins that directly control cell wall expansion and cell elongation. Here we detected the expansin-related gene expression under IAA and NPA treatment. The gene expression trend of *Expansin-B2*, *Expansin-B7,* and *Expansin-B8* at 96 h after germination in MS24B and Z210 is nearly the same. Compared with CK, the gene expression of these three expansin-related genes increased by 12.0%,533.3%, and 78.0% compared with the IAA application, respectively, while the gene expression decreased dramatically with the NPA application (*p* < 0.05), which nearly had no expression of *Expansin-B2* and *Expansin-B7* (Figure 8A). In addition, the boost of the *Expansin-B2* and *Expansin-B8* expression under IAA treatment in Z210 was higher than that in MS24B. The gene expression increase reached nearly six, seven, and five folds compared with the control in *Expansin-B2*, *Expansin-B7,* and *Expansin-B8* under IAA treatment in Z210, respectively (Figure 8B). In addition, these gene expressions in NPA treatment also decreased sharply to 76.0%, 80.0%, and 58.1% of the expression of CK treatment. Overall, with the application of IAA, the activity of H^+^-ATPase and the expansin-related gene expression level were induced to increase significantly (*p* < 0.05), which led to the membrane acidification and cell elongation in mesocotyl. The trend was the opposite for the samples treated with NPA.

**Figure 9:**
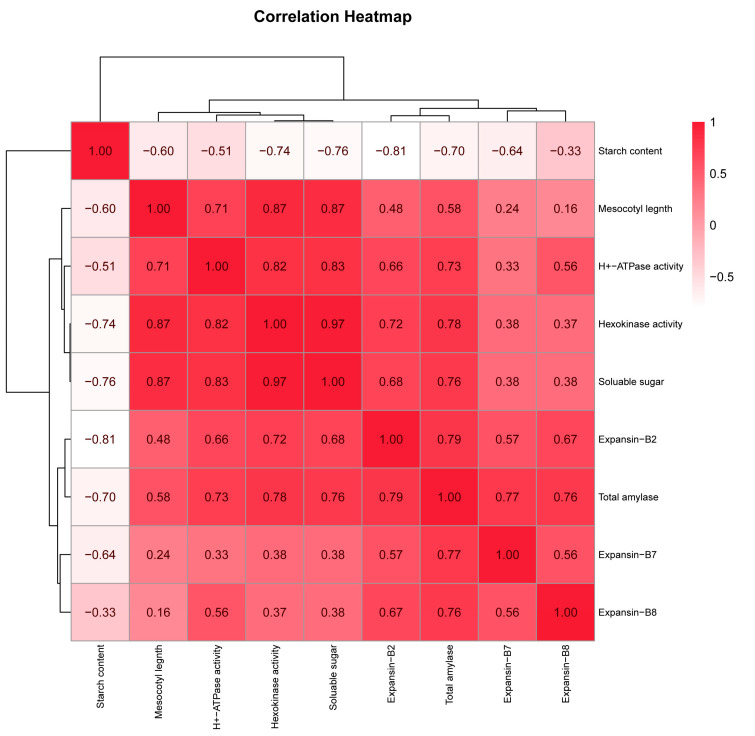
The correlation heatmap among characters measured in this study. The colors shown in the color bars, gradually from red to white, present the correlation between the two characters from high to low, respectively.

A correlation heatmap was presented to analyze the correlation of the characters measured in this research integrally. According to the results, it is shown that the mesocotyl length increase was positively correlated with the total amylase activity, soluble sugar content, hexokinase activity, H^+^-ATPase activity, and the gene expression of *Expansin-B2*, *Expansin-B7,* and *Expansin-B8*, while it had negative correlations with the starch content. The soluble sugar content and hexokinase activity had the highest correlation with mesocotyl length (Figure 9).

## 3. Discussion

In this research, two inbred sorghum lines with significantly different mesocotyl lengths were selected as experimental material. The long mesocotyl line Z210 is about five times longer than the short mesocotyl line MS24B. Deep sowing is normally practiced for crops to get more moisture for germination in drought land or improve their lodging ability. However, its disadvantage is to affect the germination rate. This problem can be conquered by planting long mesocotyl inbred lines or utilizing techniques to increase the mesocotyl length. According to our search, exogenous IAA application can promote sorghum mesocotyl elongation no matter in the long mesocotyl line or the short one. The short mesocotyl inbred presented a more obvious elongation phenotype with exogenous IAA application. Reports about IAA application on other species identified similar results. For example, Feng et al. [23] reported that the rice mesocotyl could be triggered by exogenous IAA for length elongation at 2 days after germination in darkness. In addition, another research also proved that endogenous IAA could increase by 14.7% of mesocotyl length in maize inbred line 3681-4 [24]. Furthermore, it is shown that the elongation of sorghum mesocotyl mainly depended on the elongation of cell length. Moreover, the middle part of the mesocotyl cell had the most considerable increase (*p* < 0.01) (Figure 2 and Figure 3). As we know, the top part of mesocotyl is the most active region of cell division, and the bottom part is normally the mature cell. Therefore, the middle part of mesocotyl increased significantly in the treatment of IAA. With similar results, Taiz [9] indicated that the essence of mesocotyl elongation is the increase in the cell length of mesocotyl. Z210 showed an early germination start than MS24B. Exogenous IAA did not change the start time of germination in each line. However, the NPA treatment did delay the germination time in both Z210 and MS24B. It is possible that the endogenous IAA, which NPA application can be inhabited, plays a major role in seed germination time. Miransari et al. [25] reviewed that the hormonal signaling of IAA has been related to the effects of seed gene expression on regulating seed activities during seed germination. 

A large amount of energy was needed for the germination of seed and elongation of mesocotyl. Storage materials such as starch are the primary energy source before photosynthesis. During the germination stage, starch can be hydrolyzed to soluble sugar and then offer energy for mesocotyl growth by its further decomposition. In our result, the exogenous IAA promoted the activity of amylase increase in short mesocotyl line MS24B, which then started a series of processes, including a starch content decrease and soluble sugar content increase. In addition, with the increase of the activity of hexokinase, the soluble sugar was consumed to produce more energy (Figure 5A,C,E). In Z210, the trend and value of IAA treatment and CK were nearly the same (Figure 5B,D,F). However, which was interesting in our result was that although the total starch content of Z210 was less than that of MS24B, the total amylase activity, the soluble sugar content, the hexokinase activity, and the PM H^+^-ATPase activity were higher in Z210 in CK treatment. In addition, the total amylase activity was higher at the beginning of the germination period and higher at each time point in Z210 compared with that in MS24B in CK treatment (Figure 5C,D). It means that the seeds had relatively higher starch decomposition efficiency (energy utilization efficiency) in Z210. This may be the reason why Z210 had longer mesocotyl. Moreover, the IAA application in MS24B significantly increased the total amylase activity, the soluble sugar content, the hexokinase activity, and the PM H^+^-ATPase activity, which may speed up the decomposition from starch to a series of following pathways. In other words, the exogenous IAA helped MS24B to increase the energy metabolism efficiency, which in turn, offered more energy for cell wall acidification and cell growth, which then resulted in the elongation of mesocotyl. Therefore, IAA can be potentially used as a seed coating component to increase the sorghum mesocotyl length during the germination stage. IAA also can be used as a priming material for seeds to improve the emergence performance. 

Based on the acid-growth theory, H^+^ was activated and extrusion by the PM H^+^-ATPase into the cytoplasm, which then changes intracellular pH. This acid environment led to the activation of many enzymes and expansin proteins. The final result of loosening the cell wall provides space for cell growth [26]. In Z210, it was found that exogenous application of IAA activated the expression of *expansin-B2*, *B7,* and *B8*, while the mesocotyl length had no significant changes (Figure 8B). The reason for this may be because of the crucial role of PM H^+^-ATPase, which had no dramatic changes after the application of IAA (Figure 7). Cleland [27] proved that IAA promoted cell growth by increasing cell wall sketching, which in turn, increased cell wall plasticity. Moreover, according to the research from Li et al. [28], exogenous IAA promotes cell elongation growth by the improvement of cell plasticity. In addition, it then promoted the extension of cell walls. Rober et al. [29] identified that PM H^+^-ATPase was evaluated as a downstream target of IAA, which promoted cell membrane acidification. Another research proved that IAA induces proton extrusion out of the ectoplasmic space by stimulating PM H^+^-ATPase, which then causes cell wall acidification and plasma membrane hyperpolarization to relax the cell wall and promote cell expansion [30]. In addition, Takahashi et al. [31] identified that IAA activated the PM H^+^-ATPase by phosphorylation during hypocotyl elongation. In our results, the elongation of mesocotyl cells under exogenous IAA application is the result of cell membrane acidification, which is regulated by a positive increase in the activity of PM H^+^-ATPase and the expression of Expansin. The correlation results also proved this aspect. Expansin is one type of plant cell wall relaxin, which plays a significant role in plant growth [32]. For example, *Expansin-A5*, *B3,* and *B7* were reported to be induced by IAA to regulate rice growth [33]. Another research claimed that overexpression of the expansin gene *OsEXP4* in rice would increase 97% of the mesocotyl length, and suppressing this gene would result in a 43% decrease in mesocotyl length [34]. In our results, the expression of *Expansin-B2*, *B7,* and *B8* was increased significantly after IAA treatment in both MS24B and Z210.

There were studies about IAA promoting mesocotyl elongation in other crops. However, our study brought out some new ideas. Sorghum is usually considered a pioneer crop for marginal lands. Mesocotyl plays a considerable role in the research of sorghum seedlings’ emergency and population construction. From a more practical and applied point of view, how to regulate mesocotyl growth effectively or discover potential cultivation technique has more practical value. Our study elucidated the response to sorghum mesocotyl length change through the exogenous application of IAA and its inhibitors and explained the mechanism of exogenous IAA regulation of sorghum mesocotyl length from the perspective of energy metabolism. Furthermore, the experimental materials we used in this study were different in the length of mesocotyl, and they had different reflections to the exogenous IAA application, which was that the short mesocotyl sorghum line was more sensitive to exogenous IAA application and presented a significant increase of mesocotyl length. From the results, the long and short mesocotyls of sorghum also have their own characteristics in the germination process. These results provide a material basis and research clues for deeper physiological and molecular research in the future. Based on this study, we can discover and integrate more effective means of production regulation so as to improve the emergence rate of sorghum planted in the field under adversity conditions (such as deep sowing conditions) and contribute to ensuring the yield of sorghum, which has certain theoretical and practical significance.

## 4. Materials and Methods

### 4.1. Plant Materials and Growth Conditions

Long mesocotyl sorghum (*Sorghum bicolor* L. Moench) inbred line Z210 and short mesocotyl inbred line MS24B were chosen as experiment materials. Sorghum seeds of the same size were picked to be disinfected with sodium hypochlorite (10%) and cleaned with distilled water before germination. The seeds were spread in the petri dish with moistened filter paper in plant growth chambers. The growth condition was set at 26 °C in the dark. There were three treatments of each line which encompassing the CK treatment, the IAA treatment, and the NPA treatment. The CK treatment was sowing in normal conditions with distilled water. The IAA treatment was sowing in normal conditions with 1µmol/L exogenous IAA application. The NPA treatment was sowing in the normal condition with 1µmol/L exogenous IAA and 50µmol/L exogenous NPA (IAA inhibitor) application. The mesocotyl of each treatment was sampled at a different time point after germination. 

### 4.2. Observation of Morphological Differences of Each Part of Mesocotyl

The samples for the stereomicroscope observation of CK, IAA, and NPA treatment were sampled at 96 h after germination. In Z210, 1 cm of up, middle, and down parts of mesocotyl were taken for the paraffin section made. In MS24B, the short mesocotyl was trisected into up, middle, and down parts for the paraffin section made [35]. The morphological differences of different treatments and parts were observed by stereomicroscope (Leica DVM6, Wetzlar, German). 

### 4.3. Starch and Soluble Sugar Content Measurement 

0.1 g dried seeds sample was weighed and extracted in 4 mL 80% ethanol at 80 °C for 20 min. The extraction solution was centrifuged at 4000 rpm for 5 min. The supernatants were moved to a 25 mL volumetric flask. The extraction step was repeated two times at 80 °C for 10 min. All supernatants were fixed to 25 mL and mixed gently. 5 mL ethanol extraction was quickly added to 2 mL distilled water and 6.5 mL anthrone reagent following fast shaking for 2–3 s and colored at room temperature for 10–15 min. Absorbance of the solution was measured at 620 nm wavelength. The amount of soluble sugar content was calculated by the formula below [36]:

Soluble sugar content (%) = (C × V)/(W × 106) × 100%

The remaining pellets from the centrifuge step were added to 2 mL distilled water and boiled for 15 min to evaporate the ethanol. After cooling down to room temperature, 2 mL cold 9.2 mol/L HClO_4_ was added to the solution for 10 min extraction. 4 mL distilled water was added before the 10 min centrifuge (4000 rpm). The supernatants were extracted, and the above steps were repeated two times. All supernatants were fixed to 50 mL with distilled water. A 2.5 mL solution was added to a 6.5 mL anthrone reagent with fast shaking. The measurement of absorbance was at 620 nm before standing at room temperature for 10–15 min. The amount of starch content was calculated by the formula below [37]:

Starch content % = G × 0.9/DW × 100%

### 4.4. Total Amylase Activity Measurement 

Total amylase activity measurement was referenced by Tarrago et al. [38]. One gram of ground seeds sample was mixed with 10 mL 4 °C distilled water. The enzyme extract was the supernatant of the homogenate above after 30 min centrifuge. The reaction system consisted of 0.7 mL enzyme extract, 2 mL citrate buffer (0.1 mM, pH 5.0), and 0.5 mL 2% soluble starch (freshly prepared). It was stopped by adding 2 mL color reagent after a 5 min 30 °C water bath. The absorbance was measured at 540 nm.

### 4.5. Hexokinase and PM H^+^-ATPase Activity Measurement

The hexokinase activity of sample mesocotyl was measured using a hexokinase (HK) Assay Kit (Solarbio Science and Technology, Beijing, China). The absorbance of Hexokinase was measured at 340 nm, and the activity was calculated by the manufacturer’s protocol. The activity of hexokinase was expressed in U/g fresh weight, and there were three technical repetitions for each biological sample (http://solarbio.net/images/SHSJ/BC0740.pdf accessed on 10 February 2023).

ATP hydrolysis assays were referenced in the protocol of Qiu et al. [39]. The first step was to add 10–15 μg membrane proteins into the reaction medium (25 mM). The medium consisted of 25 mM Hepes-Tris (pH 6.5), 3 mM ATP, 3 mM MgSO_4_, 50 mM KCl, 1 mM Na_3_MoO_4_, 0.015% (*w*/*v*) Triton X-100, in the presence or absence of 400μM Na_3_VO_4_. The next step was to incubate the reaction for 30 min at 37 °C and then follow with the quench by the addition of 10% (*w*/*v*) (TCA). The activity of the H^+^-ATPase measured the release of Pi [40].

### 4.6. RNA Extraction and Quantitative Real-Time PCR

Total RNA was extracted using a Trizol reagent kit (Invitrogen, Carlsbad, CA, USA) according to the manufacturer’s protocol. RNA quality was assessed on an Agilent 2100 Bioanalyzer (Agilent Technologies, Palo Alto, CA, USA) and checked using RNase-free agarose gel electrophoresis. The expression of expansin-related genes *Expansin-B2*, *Expansin-B7,* and *Expansin-B8* were measured by Realtime-qPCR in treatment CK, IAA, and NPA. The mesocotyl tissue of each treatment was sampled at 96 h after germination. Each treatment had three biological repetitions, and each one had three technical repetitions. qPCR reaction system consisted of 2 µL cDNA, 10 µL 2×SYBR Green, 0.5 µL forward primer, 0.5 µL reverse primer, and 7 µL sterile water. The qPCR conditions started from a 15 min pre-denaturation at 95 °C. This was followed by a 10 s denaturation at 95 °C and a 20 s annealing at 58 °C. The next step was an extension for 60 s at 72 °C. Cycling the step from denaturation to extension 45 times. The melting curve was added after the cycles. *Sbactin* was used as a reference gene, and the relative gene expression was calculated by the 2^−△△Ct^ method. The primers used in this experiment are listed in Table 1.

### 4.7. Statistical Analysis

All experimental data were analyzed by Excel 2016. The significant differences (*p* < 0.05) in this paper were calculated by SPSS 21.0 according to Duncan’s multiple range test principle. The data presented in the graph were expressed as mean ± standard deviation.

## 5. Conclusions

**Figure 10:**
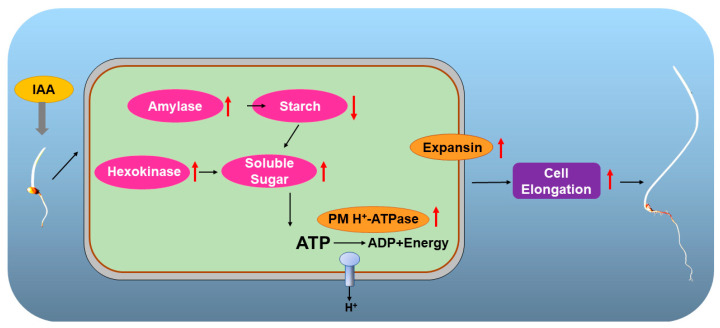
A flowchart of exogenous IAA elongate sorghum mesocotyl by energy metabolism and membrane acidification.

The exogenous IAA treatment can promote the elongation of sorghum mesocotyl length by increasing the mesocotyl cell length. It is able to accelerate the energy metabolism process. Specifically, it induced an increase in total amylase activity, which then led to a sharp drop in starch content. This is the reason for the increase in soluble sugar content. Furthermore, the increased hexokinase consumed the increased soluble sugar to produce more energy for the PM H^+^-ATPase reaction. Combining this with the increasing activity of expansins, it finally resulted in cell membrane acidification, which promoted mesocotyl cell elongation. (Figure 10).

## Figures and Tables

**Table 1:** The primers for qPCR experiment.

Gene	PCR Primer (5′-3′)
*SbActin*	ATGGCTGACGCCGAGGATATCCA GAGCCACACGGAGCTCGTTGTAG
*SbEXPB2*	GCGAGGTGAAGACGGTGATGATC GTGCCGGAGCTTGTCGTTGAG
*SbEXPB7*	CCTCCACCAGCCGTCGTCTAC CACCACCACCACTGCCGTTG
*SbEXPB8*	TAGGTAGTTGGATCGGAGCAGAGC CAGGCAGGAGAGCGAGGACAG

## Data Availability

The data that support the findings of this study are available from the corresponding author upon reasonable request.
